# Initial In Vitro and In Vivo Evaluation of a Novel CCK2R Targeting Peptide Analog Labeled with Lutetium-177

**DOI:** 10.3390/molecules25194585

**Published:** 2020-10-08

**Authors:** Anton Amadeus Hörmann, Maximilian Klingler, Maliheh Rezaeianpour, Nikolas Hörmann, Ronald Gust, Soraya Shahhosseini, Elisabeth von Guggenberg

**Affiliations:** 1 Department of Nuclear Medicine, Medical University of Innsbruck, 6020 Innsbruck, Austria; anton.hoermann@i-med.ac.at (A.A.H.); Maximilian.Klingler@i-med.ac.at (M.K.); m.rezaeianpour@yahoo.com (M.R.); 2 Pharmaceutical Chemistry and Radiopharmacy Department, School of Pharmacy, Shahid Beheshti University of Medical Sciences, 1991953381 Tehran, Iran; soraya.shahhosseini@gmail.com; 3 Department of Pharmaceutical Chemistry, University of Innsbruck, 6020 Innsbruck, Austria; nikolas.hoermann@uibk.ac.at (N.H.); ronald.gust@uibk.ac.at (R.G.)

**Keywords:** cholecystokinin-2 receptor, minigastrin, molecular imaging, targeted radiotherapy, lutetium-177

## Abstract

Targeting of cholecystokinin-2 receptor (CCK2R) expressing tumors using radiolabeled minigastrin (MG) analogs is hampered by rapid digestion of the linear peptide in vivo. In this study, a new MG analog stabilized against enzymatic degradation was investigated in preclinical studies to characterize the metabolites formed in vivo. The new MG analog DOTA-DGlu-Pro-Tyr-Gly-Trp-(*N*-Me)Nle-Asp-1Nal-NH_2_ comprising site-specific amino acid substitutions in position 2, 6 and 8 and different possible metabolites thereof were synthesized. The receptor interaction of the peptide and selected metabolites was evaluated in a CCK2R-expressing cell line. The enzymatic stability of the ^177^Lu-labeled peptide analog was evaluated in vitro in different media as well as in BALB/c mice up to 1 h after injection and the metabolites were identified based on radio-HPLC analysis. The new radiopeptide showed a highly increased stability in vivo with >56% intact radiopeptide in the blood of BALB/c mice 1 h after injection. High CCK2R affinity and cell uptake was confirmed only for the intact peptide, whereas enzymatic cleavage within the receptor specific C-terminal amino acid sequence resulted in complete loss of affinity and cell uptake. A favorable biodistribution profile was observed in BALB/c mice with low background activity, preferential renal excretion and prolonged uptake in CCK2R-expressing tissues. The novel stabilized MG analog shows high potential for diagnostic and therapeutic use. The radiometabolites characterized give new insights into the enzymatic degradation in vivo.

## 1. Introduction

Radiolabeled peptide analogs for theranostic use in the diagnosis and treatment of cancer need to fulfill important prerequisites, such as high receptor affinity, appropriate metabolic stability, high and persistent tumor uptake, as well as low uptake in non-target tissue and fast blood clearance [1]. Up to date, targeting G-protein coupled receptors overexpressed on the surface of tumor cells for nuclear medicine applications is mainly limited to radiolabeled somatostatin analogs. The cyclic somatostatin analog octreotide with high affinity to the somatostatin receptor subtype 2 is used for symptomatic and biochemical control in the treatment of neuroendocrine tumors [2]. Radiolabeled octreotide derivatives have been successfully introduced in routine nuclear medicine applications for diagnosis and treatment of neuroendocrine tumors [3]. A major milestone in this respect is the recent approval of Lutathera^®^ for peptide receptor radionuclide therapy by the European Medicines Agency (EMA) and by the Food and Drug Administration (FDA) [4,5]. So far, this success could not be translated to radiolabeled peptide analogs targeting other receptors. The reason often lies in the rapid metabolism in vivo of the linear peptide sequences derived from natural peptide hormones leading to cleavage of amino acids by enzymatic degradation and subsequent loss of affinity to the target receptor [1]. Radiolabeled peptide analogs targeting the cholecystokinin-2 receptor (CCK2R), overexpressed in different tumors, such as small cell lung cancer, stromal ovarian cancers, gastrointestinal stromal tumors, astrocytoma and especially medullary thyroid carcinoma (MTC), have shown to be very promising for application in diagnosis and therapy [6,7]. The reported clinical use has mainly focused on the diagnosis and treatment of patients with advanced MTC [8]. In the last decades, several attempts have been made to develop a radiolabeled CCK2R targeting peptide analog with suitable pharmacological properties for theranostic applications. First prove of principle studies with a radioiodinated gastrin analog confirmed the feasibility of CCK2R targeting [9]. Different CCK2R-targeting peptide analogs conjugated to the bifunctional chelators diethylenetriaminepentaacetic acid (DPTA) and 1,4,7,10-tetraazacyclododecane-1,4,7,10-tetraacetic acid (DOTA) radiolabeled with trivalent radiometals have been developed and evaluated in clinical studies [8]. The MG analog DTPA-DGlu-Glu-Glu-Glu-Glu-Glu-Ala-Tyr-Gly-Trp-Met-Asp-Phe-NH_2_ (DTPA-MG0), derived from human MG, displayed a high renal uptake hindering the therapeutic use [10]. With the removal of the penta-Glu sequence in the truncated MG analog DOTA-DGlu-Ala-Tyr-Gly-Trp-Met-Asp-Phe-NH_2_ (DOTA-MG11), the renal uptake was efficiently reduced, however, so was the stability in vivo [11,12]. Thus, the clinical applicability of the peptide analogs developed so far is limited.

CCK2R-targeting peptide analogs are potential substrates of various enzymes such as the angiotensin converting enzyme (ACE), neutral endopeptidase (NEP), aminopeptidase A (APA) and cathepsins [13,14,15,16,17]. Extensive preclinical research was undertaken to improve the stability in vivo as well as the targeting properties. Different modifications were introduced in the linear peptide sequence of different CCK2R targeting peptide analogs, such as the incorporation of unnatural amino acids, inversion of the configuration of amino acids, cyclisation of the linear peptide and dimerization [18,19,20,21]. Most of these developments have not led to the required improvements necessary for successful clinical application. Two MG analogs, DOTA-(DGlu)_6_-Ala-Tyr-Gly-Trp-Met-Asp-Phe-NH_2_ (PP-F11) labeled with indium-111 as well as DOTA-(DGlu)_6_-Ala-Tyr-Gly-Trp-Nle-Asp-Phe-NH_2_ (PP-F11N) labeled with lutetium-177, which are derived from MG0 by inversion of the configuration of the penta-Glu motif, are currently examined in clinical studies (ClinicalTrials.gov Identifier: NCT03246659 and NCT02088645) [22,23,24]. Besides chemical modification of the peptide, in situ stabilization by co-injection of enzyme inhibitors was investigated. For ^111^In-labeled DOTA-MG0 and DOTA-MG11, the use of phosphoramidone improved the tumor uptake, whereas for DOTA-MG0, a concomitant increase of renal retention occurred [25]. Sauter et al. studied the effect of two NEP inhibitors, phosphoramidone and thiorphan, on the targeting properties of ^177^Lu-labeled DOTA-MG11, PP-F11 and PP-F11N. Only for DOTA-MG11 an improved tumor uptake could be achieved, whereas no improvement was found for PP-F11 and PP-F11N [14]. The results suggest that in situ stabilization is highly dependent on the individual radiopeptide and cannot be generalized. Besides that, the long-term use of protease inhibitors, especially NEP inhibitors, can potentially cause side effects that are not yet well understood [26].

In our recent studies we could introduce new modifications within the C-terminal sequence Trp-Met-Asp-Phe-NH_2_, known to be essential for CCK2R binding [27,28,29]. Most favorable properties were found for the new MG analog with the sequence DOTA-DGlu-Ala-Tyr-Gly-Trp-(*N*-Me)Nle-Asp-1Nal-NH_2_ (DOTA-MGS5), in which methionine is replaced by *N*-methylated norleucine ((*N*-Me)Nle) and phenylalanine by 1-naphtylalanine (1Nal) [28]. Besides leading to highly improved stability in vivo, the introduced modifications also led to an enhanced receptor-specific cell uptake, as well as to a highly increased tumor uptake, when radiolabeled with different radiometals. In nude BALB/c mice bearing CCK2R-expressing tumor xenografts, a tumor uptake of more than 20% of the injected activity per gram (IA/g) was observed. This corresponds to a three-fold improvement compared to PP-F11 and PP-F11N (~6.7% and 6.9% IA/g) [14,28]. Nevertheless, some degradation products were still detected by radio-HPLC analysis of blood obtained from mice injected with ^177^Lu-labeled DOTA-MGS5 [28]. 

With the aim of further improving the in vivo stability of DOTA-MGS5, we have explored additional modification of the peptide sequence. The amino acid proline (Pro) is a promising candidate for amino acid exchange and forms a tertiary amide bond which similarly to *N*-methylated peptide bonds and triazoles may improve the stability in vivo [27,30]. In this study, a preliminary preclinical characterization of the new MG analog DOTA-DGlu-Pro-Tyr-Gly-Trp-(*N*-Me)Nle-Asp-1Nal-NH_2_ (**1**) was carried out focusing on the enzymatic stability of the ^177^Lu-labeled radiopeptide in vivo. For this purpose, besides characterizing the stability in vitro in different media, metabolic stability studies were carried out in BALB/c mice giving first insights into the enzymatic degradation and biodistribution profile of this new radiolabeled MG analog. To characterize the ^177^Lu-labeled metabolites formed in vivo, different possible metabolites of **1** were synthesized. Furthermore, the receptor affinity of the new MG analog, as well as selected metabolites, was studied in A431 human epidermoid carcinoma cells stably transfected with human CCK2R (A431-CCK2R). The same cell line was used to investigate the cell uptake of the ^177^Lu-labeled peptide analog and selected radiometabolites. Mock-transfected A431 cells (A431-mock) were used as negative control.

## 2. Results

### 2.1. Peptide Synthesis and Radiolabeling 

**Figure 1:**
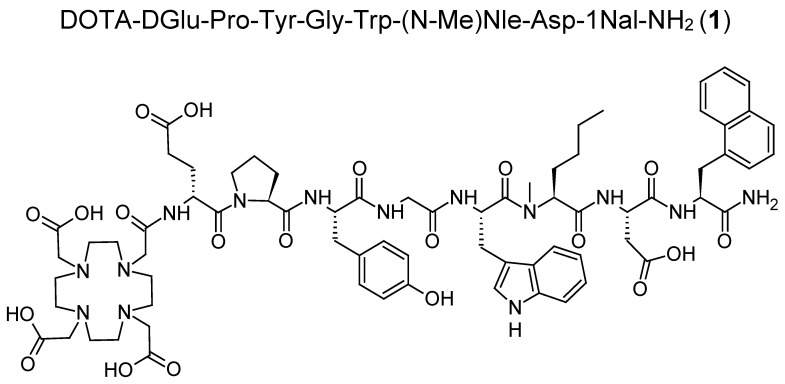
Amino acid sequence and chemical structure of **1**.

The amino acid sequence and chemical structure of **1** is displayed in Figure 1. **M1**–**M6** were synthesized by standard solid phase peptide synthesis starting from 100 mg of resin following the synthesis protocol described below. For **M7** and **M8,** which were synthesized by different strategies, conjugation with DOTA (DOTA-tris(tert-butyl) ester or DOTA mono-*N*-hydroxysuccinimide ester) was carried out in solution. After purification by reversed phase HPLC (RP-HPLC), characterization by mass spectrometry and lyophilization, the metabolites **M1**–**M8** were obtained with a purity >95% (with the exception of **M1** and **M4,** for which a purity of 93–94% was achieved). The amino acid sequences and analytical data for **1** and the different metabolites **M1**–**M8** are shown in Table 1.

**Figure 2:**
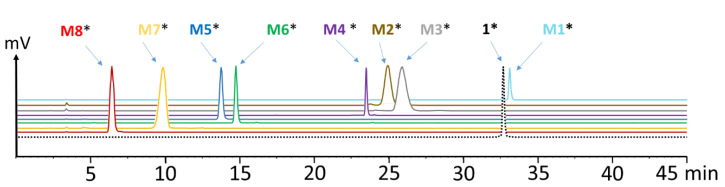
Radio-HPLC chromatograms of [^177^Lu]Lu-**1** and its ^177^Lu-labeled metabolites **M1**–**M8** (* indicating radiolabeled with lutetium-177).

For experiments in vitro, labeling with lutetium-177 was carried out at a low molar activity of 10–20 MBq/nmol, yielding nearly quantitative labeling and allowing the use of the radiolabeled conjugates without further purification. The radiolabeled conjugates used in animal studies were radiolabeled at a higher molar activity of ~40 MBq/nmol. Hydrophilic impurities were removed by solid phase extraction (SPE) to obtain the radiolabeled peptides with a radiochemical purity of >99%. The radio-HPLC chromatograms of the radiolabeled compounds are shown in Figure 2.

### 2.2. Characterization In Vitro

**Figure 3:**
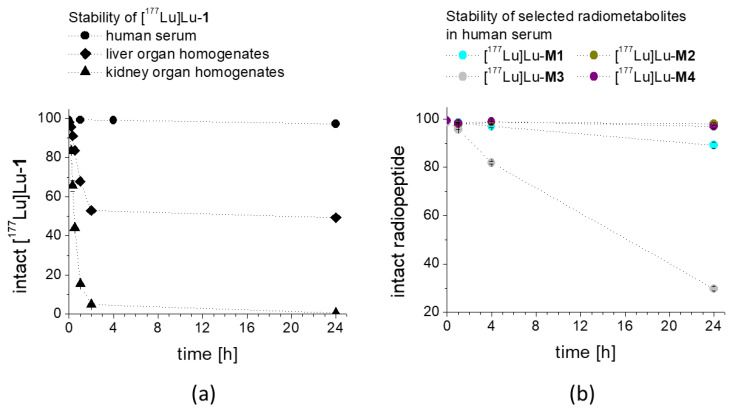
Stability of (**a**) [^177^Lu]Lu-**1** after incubation in human serum as well as in rat liver and rat kidney homogenates, and of (**b**) selected radiolabeled metabolites **M1**–**M4** in human serum, as analyzed up to 24 h after incubation.

The stability of ^177^Lu-labeled **1** and **M1**–**4** in fresh human serum, as well as the stability of [^177^Lu]Lu-**1** in liver and kidney homogenates, was analyzed for up to 24 h after incubation. The percentage of intact radiopeptide found over time is presented in Figure 3. [^177^Lu]Lu-**1** showed a very high stability in human serum with values of 96.2 ± 1.3% intact peptide after 24 h incubation. A higher degree of degradation was found in liver homogenate with >90% intact radiopeptide up to 20 min after incubation and decreasing to 83.6 ± 0.2, 52.9 ± 2.0 and 49.3 ± 1.7%, at 30 min, 2 h and 24 h, respectively. In kidney homogenate, a faster metabolic breakdown was observed. However, still 65.6 ± 2.8 and 43.9 ± 1.0% intact radiopeptide were present 20 min and 30 min after incubation. At later time points, the radiolabeled conjugate was completely degraded (<5 and <1% at 2 and 24 h after incubation, respectively). In human serum, a high stability was also found for [^177^Lu]Lu-**M1** (89.1 ± 1.3%), [^177^Lu]Lu-**M2** (98.1 ± 0.3%) and [^177^Lu]Lu-**M4** (98.4 ± 0.01%) after 24 h incubation in human serum, whereas [^177^Lu]Lu-**M3** showed a different behavior. A fast enzymatic degradation occurred in serum with only 29.7 ± 0.8% of intact [^177^Lu]Lu-**M3** 24 h after incubation.

The logD values calculated from the octanol/PBS distribution of the different ^177^Lu-labeled peptides resulted in a hydrophilicity profile in the order of [^177^Lu]Lu-**M4** (−4.19 ± 0.30) > [^177^Lu]Lu-**M2** (−4.18 ± 0.17) > [^177^Lu]Lu-**M3** (−4.13 ± 0.11) > [^177^Lu]Lu-**M1** (−4.07 ± 0.35) > [^177^Lu]Lu-**1** (−1.96 ± 0.07). 

Protein binding in human serum as analyzed by size exclusion chromatography was found to be lowest for [^177^Lu]Lu-**M3** (23.4 ± 1.9%), followed by [^177^Lu]Lu-**1** (37.8 ± 2.9%), [^177^Lu]Lu-**M4** (41.7 ± 0.2%), [^177^Lu]Lu-**M1** (45.4 ± 1.8%) and [^177^Lu]Lu-**M2** (48.1 ± 2.3%) for the time point of 24 h after incubation.

### 2.3. Cell Internalization and Receptor Binding Studies

**Figure 4:**
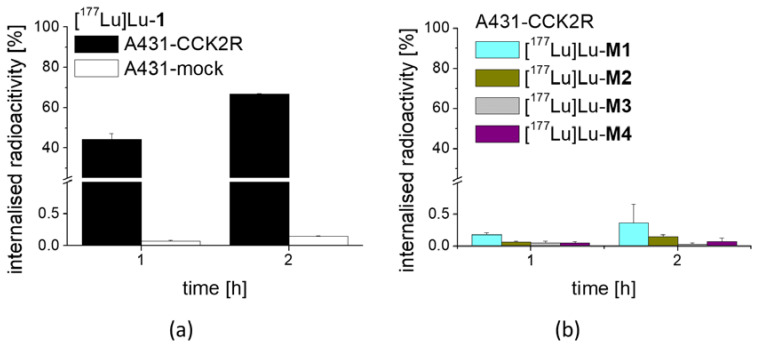
Cell uptake of (**a**) [^177^Lu]Lu-**1** in A431-CCK2R and A431-mock cells and (**b**) ^177^Lu-labeled **M1**–**M4** in A431-CCK2R cells, after 1 h and 2 h incubation.

For [^177^Lu]Lu-**1** incubated with A431-CCK2R cells, a high internalization with uptake values of 44.4 ± 2.7% after 1 h incubation (Figure 4) was observed. As expected, the ^177^Lu-labeled metabolites showed no internalization into A431-CCK2R cells ([^177^Lu]Lu-**M1**: 0.18 ± 0.03%, [^177^Lu]Lu-**M2**: 0.06 ± 0.02%, [^177^Lu]Lu-**M3**: 0.05 ± 0.03% and [^177^Lu]Lu-**M4**: 0.05 ± 0.02%). Additionally, after 2 h incubation a very high receptor-specific uptake could be confirmed for [^177^Lu]Lu-**1**, with values further increasing to 66.6 ± 0.3%, whereas no internalization occurred for the ^177^Lu-labeled metabolites. Specificity of the cell uptake was proven by contemporaneous incubation in A431-mock cells finding a cell uptake of <0.2% and <0.4% for [^177^Lu]Lu-**1** at 1 and 2 h, respectively.

**Figure 5:**
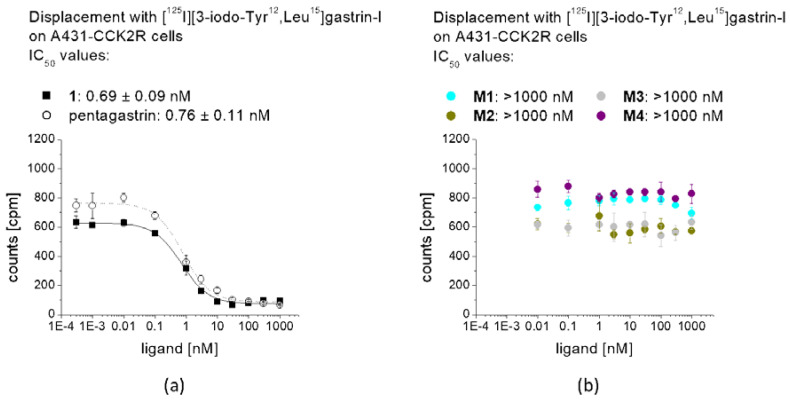
Competitive binding curves against [^125^I][3-iodo-Tyr^12^,Leu^15^]gastrin-I for (**a**) non-labeled **1** in comparison with pentagastrin, as well as (**b**) non-labeled **M1**–**M4.**

In competition assays against [Leu^15^]gastrin-I substituted with iodine-125, a high binding affinity to CCK2R was confirmed for **1** (IC_50_: 0.69 ± 0.09 nM) on A431-CCK2R cells, comparable to pentagastrin used as a reference (IC_50_: 0.76 ± 0.11 nM) (Figure 5). For **M1**–**M4** no binding affinity could be observed, confirming the complete loss of receptor binding after removal of the C-terminal amide function and the C-terminal amino acids (*N*-Me)Nle, Asp and 1Nal. Based on these findings, the cell uptake and receptor binding of the remaining metabolites were not tested.

### 2.4. Stability In Vivo and Biodistribution Studies

**Figure 6:**
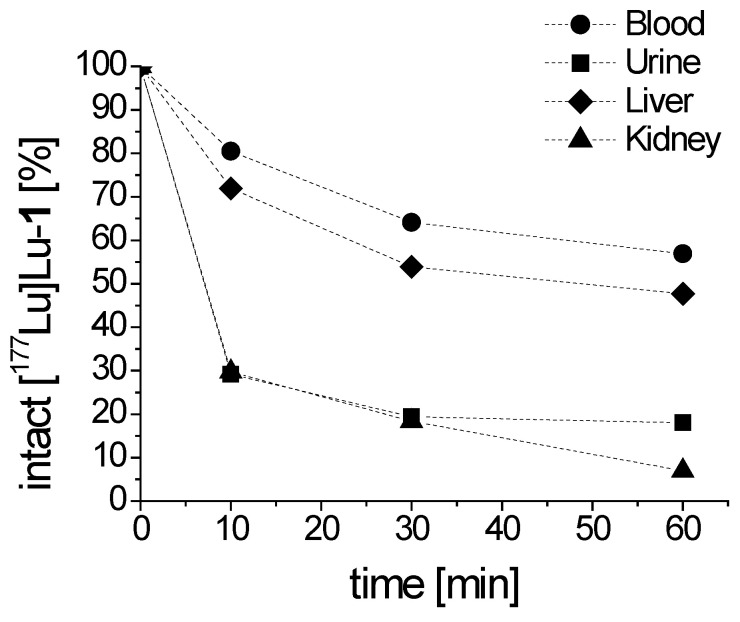
Intact [^177^Lu]Lu-**1** detectable in blood and urine, as well as liver and kidney homogenates obtained from BALB/c mice, as analyzed up to 60 min p.i.

The metabolic stability in vivo, as shown in Figure 6, was monitored after intravenous injection of [^177^Lu]Lu-**1** in BALB/c mice. A high resistance against enzymatic degradation was found at 10 min post injection (p.i.) with 80.5% intact radiopeptide present in blood. A high percentage of intact radiopeptide in blood was also observable after 30 and 60 min p.i. with values of 64.1% and 56.9%, respectively. Analysis of the urine of the mice showed that only 29.1%, 19.4% and 18.0% intact [^177^Lu]Lu-**1** was excreted at the different time points studied. In the soluble phase extracted from liver homogenate, 71.9%, 53.9% and 47.7% intact peptide were found after 10, 30 and 60 min p.i., respectively, reflecting the stability of the radiolabeled conjugate during circulation. Metabolism during excretion was confirmed by the analysis of the soluble phase extracted from kidney homogenate with values of 29.7%, 18.4% and 7.0% intact radiopeptide after 10, 30 and 60 min p.i., respectively. 

**Figure 7:**
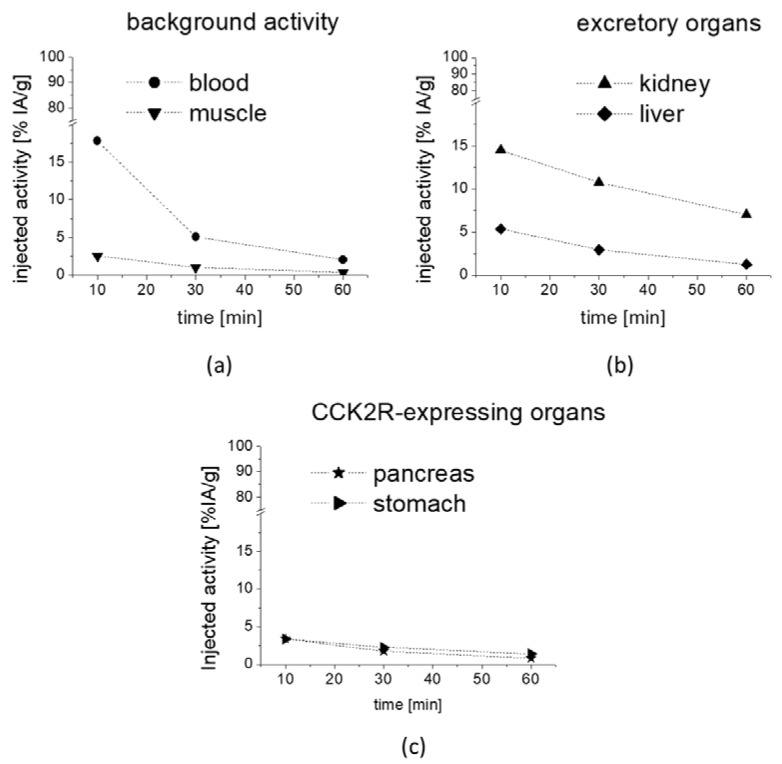
Uptake values in selected tissues as obtained from metabolic biodistribution studies with [^177^Lu]Lu-**1** in BALB/c mice at 10, 30 and 60 min p.i.: (**a**) background activity in blood and muscle, (**b**) excretory organs kidney and liver as well as (**c**) CCK2R-expressing pancreas and stomach. Values are expressed as % IA/g.

The preliminary evaluation of the biodistribution profile of [^177^Lu]Lu-**1** showed a fast clearance from the blood with low non-specific uptake in most tissues. The observed uptake values are summarized in Table 2. The whole body activity ranged from 61.78% IA at 10 min p.i. to 12.27% IA at 1 h after injection. As shown in Figure 7a, the blood pool activity rapidly declined from 17.79% to 2.05% IA/g at 10 and 60 min, respectively. Consequently, a very low background activity was found in muscle with values ranging from 2.56% to 0.36% IA/g at 10 min and 60 min, respectively. Low non-specific uptake and rapid washout was observed also for lung (17.45% to 1.95% IA/g), heart (7.13% to 0.99% IA/g), femur (5.97% to 0.52% IA/g) and spleen (3.70% to 0.79% IA/g). Excretion occurred mainly through the kidneys with activity values decreasing from 14.49% to 7.01% IA/g from 10 to 60 min p.i. (Figure 7b). The activity values observed in liver (5.37% and 1.26% IA/g) and intestine (2.63% and 1.02% IA/g) for the same time points were much lower and comparable to the non-specific uptake in other organs. When looking at the washout of radioactivity from different tissues, a higher retention of radioactivity was observed for CCK2R-expressing stomach and pancreas, which was more prominent for stomach. The percentage decrease of radioactivity at 1 h versus 10 min p.i. was lower for stomach (59%) and pancreas (75%) in comparison to the washout of 79–91% from non-excretory organs (blood, lung, heart, femur, spleen and muscle), indicating a receptor-specific uptake in stomach and pancreas (Figure 7c, Table 2). No further blocking studies were performed to investigate the specificity of the uptake in more detail.

### 2.5. Identification of the Radiometabolites Formed In Vivo

**Figure 8:**
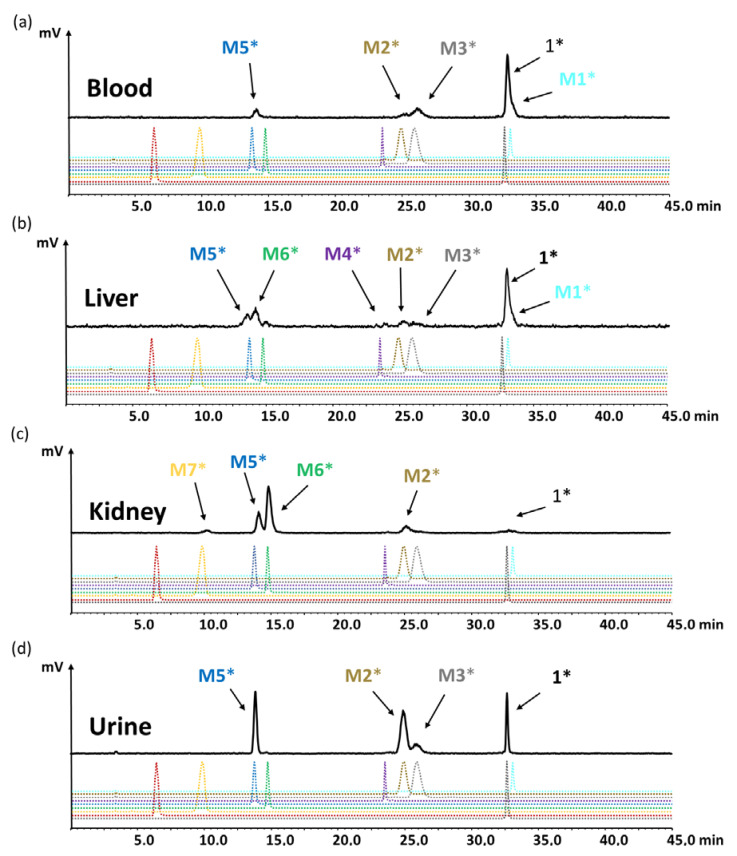
Radiochromatograms obtained from (**a**) blood, (**c**) liver, (**d**) kidneys and (**b**) urine of BALB/c mice injected with [^177^Lu]Lu-**1** for the time point of 60 min p.i.; radiochromatograms of ^177^Lu-labeled peptides **1** and **M1**–**M8** are shown for comparison (* indicating radiolabeled with lutetium-177).

For the evaluation of the radiometabolites formed in vivo, blood and urine collected from BALB/c mice injected with [^177^Lu]Lu-**1** were analyzed by radio-HPLC. In addition, the soluble phase extracted from kidney and liver homogenates was analyzed. [^177^Lu]Lu-**1** showed a high stability in blood against enzymatic degradation in vivo with a total amount of radiometabolites of 19.5%, 35.9% and 43.1% at 10, 30 and 60 min p.i., respectively. The radiometabolites observed could be matched to [^177^Lu]Lu-**M1**, [^177^Lu]Lu-**M2**, [^177^Lu]Lu-**M3**, [^177^Lu]Lu-**M5**, with hydrolysis of the C-terminal amide, as well as cleavage of the peptide bonds of Asp-1Nal, (*N*-Me)Nle-Asp and Gly-Trp. The same metabolites were also observed in urine, whereas the presence of [^177^Lu]Lu-**M1** was negligible. However, much higher levels of the radiometabolites were observed in urine, which was to be expected, given the renal pathway as main route of excretion. A similar pattern was observed also for liver and kidneys, where only minor additional radiometabolites could be detected. [^177^Lu]Lu-**M6** with cleavage of the peptide bond of Tyr-Gly was confirmed for both, liver and kidneys, while [^177^Lu]Lu-**M4** cleaved between Trp and (*N*-Me)Nle was detectable only in liver and [^177^Lu]Lu-**M7** cleaved between Pro and Tyr only in kidneys. No cleavage between DGlu and Pro was observed, confirming a stabilizing effect for the tertiary amide bond introduced into the peptide backbone. Radiochromatograms of the different samples analyzed are displayed in Figure 8a–d. The percentage of the intact radiopeptide and of the different radiometabolites found for the different time points p.i. are summarized in Table 3. 

## 3. Discussion

The strategy of CCK2R targeting with radiolabeled gastrin analogs for diagnostic and therapeutic application in patients with advanced tumors, in particular MTC, has been pursued for more than two decades. The successful clinical use of radiolabeled somatostatin analogs for targeting somatostatin receptors in patients with neuroendocrine tumors could not yet be translated to radiolabeled minigastrin analogs targeting CCK2R. In our recent studies, we could develop a new stabilization strategy leading to increased stability of the linear peptide sequence against enzymatic degradation in vivo and improving the targeting properties [28,29]. In this study, we have further explored our stabilization strategy by introducing an additional modification in the *N*-terminal region of the peptide backbone in our lead compound DOTA-MGS5. Alanine in position 2 was replaced by proline leading to the new peptide analog **1**. Furthermore, different possible metabolites were synthesized and analyzed in comparative studies with the aim to further investigate the enzymatic degradation in vivo. Pro was selected as a promising candidate for substitution, as the cyclic structure of the side chain with its conformational rigidity may protect the peptide against enzymatic degradation. The insertion of Pro into proteins influences the formation of α-helices and ß-sheets in dependence of the molecular environment conferring specific features to protein structure and folding [31]. It has been shown that the single change from the l- to d-configuration of the Glu residues in MG analogs alters the secondary structure of the peptide leading to improved serum stability [32].

In this study, the preclinical properties of [^177^Lu]Lu-**1** were evaluated and the metabolites thereof formed during degradation in vivo were characterized to explore additional possible stabilization strategies in the development of CCK2R targeting radiopeptides. To enable the characterization of the radiometabolites, eight different metabolites of **1** were synthesized in moderate yields. For in vitro studies, **1** and the metabolites thereof were radiolabeled with [^177^Lu]LuCl_3_ at low molar activity allowing quantitative radiolabeling at high radiochemical purity >95%. Receptor affinity assays with the non-labeled peptides as well as cell uptake studies with the peptide derivatives radiolabeled with lutetium-177 were performed using A431-CCK2R cells. The results confirmed loss of receptor affinity as well as cell uptake for different metabolites studied, while the intact peptide showed a retained high CCK2R affinity and improved receptor-mediated cell uptake of more than 60% of the total activity added. The specificity of the cell uptake was verified via lack of uptake in A431-mock cells used as a control cell line. Hydrolysis of the C-terminal amide was sufficient to cause complete loss of receptor affinity, proving that the interaction of the amidated C-terminus with the binding pocket is essential for maintaining receptor affinity [33]. When incubated in fresh human serum, used as a model in vitro to measure the resistance against enzymatic degradation, radiolabeled **1** as well as **M2** and **M4** showed the highest stability after 24 h incubation (>98%). Radiolabeled **M1** showed a slightly lower stability of 89%, whereas for **M3**, less than 30% intact radiopeptide was detectable after 24 h incubation, suggesting a high enzymatic susceptibility. This could be explained by the lower protein binding of [^177^Lu]Lu-**M3** leading to higher levels of free radiopeptide susceptible to proteases when compared to [^177^Lu]Lu-**1** and radiolabeled **M1**, **M2** and **M4**. Thus, increased protein binding may play an additive role in protecting [^177^Lu]Lu-**1** against enzymatic degradation. Highly improved stability of [^177^Lu]Lu-**1** was observed also in rat tissue homogenates in vitro. It has been shown that unsubstituted DOTA-MG11 labeled with indium-111 is highly susceptible to proteolytic digestion and rapidly and completely degraded within 30 min in liver homogenate and within 10 min in kidney homogenate [34]. For [^177^Lu]Lu-**1** still 84% and 44% intact radiopeptide could be observed in liver and kidney homogenate, respectively, after 30 min incubation. Only at the later time point of 2 h after incubation, an almost complete breakdown occurred in kidney homogenate, whereas in liver homogenate >50% intact radiopeptide was still present. The resistance against enzymatic degradation in rat liver homogenate was also improved when compared to DOTA-MGS5 labeled with different radiometals for which less than 30% intact radiopeptide was found for the same time point, suggesting a potential additive stabilizing effect of the insertion of Pro in position 2 [28]. 

Incubation in organ homogenates is connected with a higher breakdown of the radiopeptide due to the exposure to extracellular and intracellular proteases released after tissue homogenization, possibly leading to an underestimation of the stability in vivo [16]. Therefore, additional metabolic biodistribution studies in female BALB/c mice were performed to monitor the metabolites formed in vivo. After intravenous injection of [^177^Lu]Lu-**1** in BALB/c mice, a highly improved stability with more than 80% intact radiopeptide in blood was observed at 10 min after injection. The in vivo stability during circulation was tested for up to 1 h p.i. still finding 56.9% intact [^177^Lu]Lu-**1**. Much higher amounts of metabolites were present in the urine of the mice at the different studied time points, with the intact radiopeptide decreasing from 29% at 10 min to 18% at 1 h after injection. The metabolites found in liver homogenate resembled the metabolites observed in blood. The same correspondence was found for the metabolites in urine and kidney homogenate. A more rapid metabolism of the radiopeptide was observed in kidneys and urine (<30%), whereas in blood and liver, a much higher stability was observed. With the optimized radio-HPLC gradient allowing for a better separation of the different radiometabolites, possibly additional radiometabolites could be monitored, which were not detectable in previous studies with ^177^Lu-labeled DOTA-MGS5 [28]. The additional substitution with Pro did, however, not show a considerable effect on in vivo stability. Still, the in vivo stability of [^177^Lu]Lu-**1** is highly improved when compared to other MG analogs which are currently investigated in clinical trials [22,24]. For PP-F11 labeled with indium-111, the metabolic stability in the blood of mice was tested for the time point of 5 min p.i. finding >70% intact radiopeptide [28,35]. However, when analyzing the in vivo stability for a later time point, we found that ^177^Lu-labeled PP-F11 is almost completely degraded at 30 min p.i. [28].

To identify the observed degradation products, different metabolites were synthesized and radiolabeled with lutetium-177 for comparative radio-HPLC analysis. The retention times of the different radiometabolites were matched with the metabolites found in vivo. In the blood of mice at different time points of up to 1 h p.i., only minor amounts of radiolabeled **M1** (<10%), **M2** (<8%), **M3** (<21%) and **M5** (<10%) resulting from hydrolysis of the C-terminal amide, as well as cleavage of the peptide bonds of Asp-1Nal, (*N*-Me)Nle-Asp and Gly-Trp were found. The same metabolites, except **M1**, were detected in urine, with [^177^Lu]Lu-**M2** (~40%) and [^177^Lu]Lu-**M5** (~30%) being the most prominent. In liver, besides similar amounts of the metabolites found in blood, additionally, radiolabeled **M4** with cleavage of the peptide bond of Trp-(*N*-Me)Nle was detectable at very low concentration (<3%), as well as radiolabeled **M6** with cleavage between Tyr and Gly (~20%). This could reflect the higher enzymatic turnover in the liver. In kidneys, only 7% of intact radiopeptide was found at 1 h p.i. even though still 18% were present in the urine at the same time point, whereas much higher amounts of [^177^Lu]Lu-**M2** (12%), [^177^Lu]Lu-**M5** (24%) and [^177^Lu]Lu-**M6** (53%) were detected. Furthermore, minor amounts of [^177^Lu]Lu-**M7** (~4%) with cleavage of the peptide bond of Pro-Tyr were observed. Despite the different radiometabolites found, the new minigastrin analog, with 56.9% intact radiopeptide still present in the blood at 1 h after injection, shows a highly improved stability in vivo. 

It is well known from the literature that different enzymes are involved in the metabolism of members of the gastrin/CCK family such as the angiotensin converting enzyme (ACE), endopeptidase (NEP), aminopeptidase A (APA) and cathepsins. ACE is a zinc- and chloride-dependent peptidyl dipeptidase, widely distributed throughout the body including the lungs, gastrointestinal tract, vascular endothelium and blood, with broad specificity and besides inactivating vasoactive peptides also acts on other bioactive peptides [16,36]. In the degradation process of CCK and gastrin analogs with eight or less amino acids, ACE initially cleaves the amidated C-terminal dipeptide Asp-Phe-NH_2_ and releases a further C-terminal di- or tripeptide in a secondary step [13]. Thirteen amino acid long MG analogs containing the penta-Glu motif seem to be ACE-resistant [14]. It has however been reported that radiolabeled MG analogs derived from MG11 and MG0 are not cleaved by ACE and ACE inhibitors cannot prevent the degradation in vivo [14,15]. NEP is a zinc-dependent cell-surface enzyme with wide distribution in the body, including the presence on granulocytes and endothelial cells of the vasculature compartment, as well as in major organs such as liver, kidneys and gastrointestinal tract, and is involved in the degradation of many bioactive peptides [16,37]. In the degradation process of CCK and gastrin analogs, NEP cleaves the peptide at Asp-Phe, Trp-Met, Gly-Trp, as well as Ala-Tyr in the case of gastrin [38,39]. The fact that co-injection of ^111^In-labeled DOTA-MG11 together with a NEP inhibitor clearly increased the amount of intact radiopeptide in the blood of mice and led to a significant improvement of tumor uptake, points out the importance of NEP in the metabolism of radiolabeled MG analogs [15]. Incubation in vitro with neprylisin-1 and neprylisin-2 confirmed cleavage at Asp-Phe and Gly-Trp for DOTA-MG11, whereas for DOTA-PP-F11, only cleavage at Asp-Phe was observed and DOTA-PP-F11N with Met replaced by Nle seemed to be resistant against neprylisins. Interestingly, NEP inhibition did not result in improved tumor uptake of DOTA-PP-F11 and DOTA-PP-F11N [14]. APA is a membrane-bound type II zinc metalloprotease with broad tissue distribution [16,40]. It cleaves *N*-terminal glutamyl and aspartyl residues and is known to be involved in the degradation of CCK-8. *N*-terminal modification of CCK8 analogs led to resistance against APA, suggesting that also radiolabeled peptide analogs with *N*-terminal conjugation of a bifunctional chelator are APA-resistant [41]. During intracellular trafficking also other proteases may be involved in the degradation process. Cathepsins are a group of enzymes whose primarily function is to act as intralysosomal enzymes and in addition to that are involved in cancer development and progression [17,42]. When analyzing the stability against different cathepsins in vitro, cleavage sites at Asp-Phe and Met/Nle-Asp have been confirmed for different DOTA-conjugated MG analogs [14].

[^177^Lu]Lu-**M2** and [^177^Lu]Lu-**M5** with cleavage at Asp-1Nal and Gly-Trp, respectively, were present in all samples studies, indicating a major involvement of NEP in the metabolization of [^177^Lu]Lu-**1** in vivo. The presence of [^177^Lu]Lu-**M3** in almost all samples studies, as well as of [^177^Lu]Lu-**M6** in the soluble phase of the homogenates from liver and kidney, suggests that also an ACE-dependent metabolism could occur. Kolenc-Peitl et al. have also suggested two different degradation pathways for MG analogs, one pathway via ACE-like enzyme activity with cleavage at Met-Asp, Gly-Trp, Tyr-Gly and Ala-Tyr, and another pathway directly releasing gastrin-6 with cleavage at Ala-Tyr [43]. Interestingly, hydrolysis of the C-terminal amide resulting in [^177^Lu]Lu-**M1**, especially in blood and liver, was additionally observed, suggesting that other enzymes are also involved in the metabolism of [^177^Lu]Lu-**1**, which have not yet been characterized. Our results are in contrast to Sauter et al. who investigated the susceptibility of different DOTA-conjugated MG analogs against various proteases in vitro [14]. Based on the radiometabolites found in the different tissue samples obtained from BALB/c mice injected with [^177^Lu]Lu-**1,** we could not confirm ACE resistance in vivo, and also replacement of Met by Nle did not result in liability against neprylisins. However, the fact that the radiometabolites [^177^Lu]Lu-**M2** and [^177^Lu]Lu-**M3** were found in all investigated tissues supports a possible involvement of cathepsins in the degradation process. For [^177^Lu]Lu-**1**, only minor amounts of [^177^Lu]Lu-**M4** cleaved at (*N*-Me)Nle-Trp were observed in liver homogenate, indicating a stabilizing effect of the introduced *N*-methylated peptide bond. In a previous study, we could already show that single substitution with 1Nal in position 8 did not improve in vivo stability and only additional substitution with (N-Me)Nle in position 6 allowed to stabilize the linear peptide against enzymatic degradation [28,29]. When introducing 1,4-disubstituted 1,2,3-triazoles as metabolically stable bioisosteres in replacement of the amide bonds in Nle-substituted DOTA-MG11, Grob et al. also found the highest impact on the stability against proteases in human blood for the triazole insertion at Trp-Nle. The stability was not considerably further improved by additional triazole insertion at Ala-Tyr or Tyr-Gly. On the other hand, the insertion of triazoles at Tyr-Gly, Ala-Tyr, DGlu-Ala had a remarkable effect on tumor uptake, which was however clearly inferior to DOTA-MGS5 [28,30,44]. The introduction of an *N*-methylated peptide bond at Nle-Trp showed both effects of increased stability and improved receptor interaction. DOTA-MGS5 radiolabeled with different radiometals, showing the combined substitution of (*N*-Me)Nle and 1Nal in position 6 and 8, respectively, displayed a considerably higher cell uptake of >50%, when compared to ~25% observed for ^111^In-labeled DOTA-MGS1 with single replacement of Phe by 1Nal [29]. The combination of increased stability and improved receptor interaction led to a considerable improvement in tumor uptake. In mice xenografted with A431-CCK2R cells a very high tumor uptake of more than 20%IA/g in combination with improved tumor-to-kidney ratio (4–6) was observed for DOTA-MGS5 labeled with indium-111, lutetium-177 or gallium-68 [28]. In the present study, very high receptor-mediated cell uptake in A431-CCK2R cells of >60% was also found for [^177^Lu]Lu-**1** showing additional substitution with Pro in position 2. Interestingly, the radiometabolite [^177^Lu]Lu-**M8** with cleavage at DGlu-Pro was not found in any of the tissues examined, indicating a possible additional stabilizing effect of the introduction of Pro. In a previous study analyzing the blood of patients injected with ^111^In-labeled DOTA-MG11, high amounts of the short chain radiometabolites of DOTA-DGlu, DOTA-DGlu-Ala and DOTA-DGlu-Ala-Tyr were confirmed at 10 min after injection [12].

The preliminary biodistribution profile obtained for [^177^Lu]Lu-**1** from the metabolic studies in mice confirmed a rapid clearance from blood and low unspecific uptake in most tissues, together with predominant renal excretion. The prolonged retention of radioactivity in CCK2R-expressing stomach and pancreas indicates that [^177^Lu]Lu-**1** also shows high potential for targeting CCK2R-expressing tumors [45].

## 4. Materials and Methods

### 4.1. Materials

All commercially obtained chemicals were of analytical grade and used without further purification. No-carrier-added [^177^Lu]LuCl_3_ produced from highly enriched ^176^Yb was purchased from Isotope Technologies (Garching, Germany). Dr. Luigi Aloj kindly provided the A431 human epidermoid carcinoma cell line stably transfected with the plasmid pCR3.1 containing the full coding sequence for the human CCK2R, as well as the same cell line transfected with the empty vector alone [46]. A431-CCK2R and A431-mock were cultured in Dulbecco’s Modified Eagle Medium (DMEM) supplemented with 10% (*v/v*) fetal bovine serum and 5 mL of a 100× penicillin-streptomycin-glutamine mixture at 37 °C in a humidified 95% air/5% CO_2_ atmosphere. Media and supplements were purchased from Invitrogen Corporation (Lofer, Austria). **1** was purchased from piCHEM (Raaba-Grambach, Austria).

### 4.2. Peptide Synthesis

The different metabolites of **1**, namely **M1**–**M6** shown in Table 1, were synthesized using 9-fluorenylmethoxycarbonyl (Fmoc) chemistry. The peptides were assembled on 2-chlorotritylchloride (2-CTC) resin with capacity 1.6 mmol/g (Iris Biotech GmbH, Marktredwitz, Germany). The reactive side chains of the amino acids were masked with the following protection groups: tert-butyl ester for Asp and DGlu, tert-butyl ether for Tyr, and tertbutyloxycarbonyl (BOC) for Trp. All coupling reactions were performed using a 5-fold excess of Fmoc-protected amino acids, 1-hydroxy-7-aza-benzotriazole (HOAt) and *O*-(7-Azabenzotriazole-1-yl)-*N*,*N*,*N*′,*N′*-tetramethyluronium hexa-fluorophosphate (HATU) in dimethtylformamide (DMF) and pH adjusted to 8 with *N*,*N*′-diisopropylethylamine (DIPEA). The resin was loaded with 30% of total capacity and the remaining binding sites were capped with methanol/DIPEA/dichloromethane (DCM) in a ratio of 200 µL/100 µL/2 mL for 30 min at room temperature. Between every conjugation step, the product was washed 6 times with DMF for 1 min. Removal of the Fmoc protecting groups was obtained by two consecutive treatments with 5 mL of 20% piperidin in DMF for 5 and 15 min each. For the coupling of DOTA, a 3-fold molar excess of DOTA-tris(tert-butyl ester), HOAt and HATU was used. Cleavage of the peptides from the resin with concomitant removal of acid-labile protecting groups was achieved by treatment with a mixture of trifluoroacetic acid (TFA), triisopropylsilane, and water in a ratio of 95/2.5/2.5 (*v/v*/*v*). The crude peptides were precipitated in ice-cold ether before HPLC purification and characterized by analytical HPLC (Dionex, Germering, Germany and matrix-assisted laser desorption/ionization time of flight mass spectroscopy (MALDI-TOF MS) (Bruker Daltonics, Bremen, Germany). Purification was performed by RP-HPLC on a GILSON 322 chromatography system with a GILSON UV/VIS-155D multi-wavelength UV detector, equipped with an Eurospher II 100-5 C18 A column, 250 × 8 mm (Knauer, Berlin, Germany) or an Eurosil Bioselect 300-5 C18 A column, Vertex Plus, 300 × 8 mm, combined with an Eurosil Bioselect 300-5 C18 precolumn, Vertex Plus A, 30 × 8 mm (Knauer, Berlin, Germany), using a water/ACN/0.1% TFA gradient.

Analytical HPLC was performed using an UltiMate 3000 chromatography system consisting of a variable UV-detector (UV-VIS at λ = 220 nm), a HPLC pump, an autosampler, a radiodetector (GabiStar, Raytest, Straubenhardt, Germany), equipped with a Phenomenex Jupiter 4 μm Proteo 90 Å C12 column, 250 × 4.6 mm (Phenomenex Ltd., Aschaffenburg, Germany) and analyzed with Chromeleon Dionex Software (Version 7.2.9.11323). The radiodetector was equipped with two different loops, a low-sensitivity loop of 5 µL and a high-sensitivity loop of 250 µL. Mass spectrometry was performed using a Bruker microflex benchtop MALDI-TOF MS with 200 shots per spot in reflector acquisition mode with a positive ion source. For mass determination, samples were prepared on α-cyano-4-hydroxycinnamic acid (HCCA) matrix using dried droplet procedure. Flex Analysis 2.4 software was used to analyze the recorded data. HPLC chromatograms and MS spectra are presented in the supporting information (Figures S1 and S2). The lyophilized peptide derivatives were stored at −20 °C.

**M7** and **M8** were obtained using other strategies. For the synthesis of **M7**, the dipeptide DGlu-Pro was synthesized on 2-CTC resin following the synthesis protocol described above defining the amino acid sequence. After cleavage from the resin, precipitation in ice-cold ether, purification by HPLC and lyophilization, DGlu-Pro (0.015 g, 0.061 mmol) was transferred to a round-bottomed flask, dissolved in 1 mL ACN and pH adjusted to 8 with 25 µL DIPEA. DOTA-*N*-hydroxysuccinimid ester (0.023 g, 0.030 mmol) was added to the solution and the mixture was stirred at room temperature overnight. After evaporation of the solvent, the product was dissolved in 2 mL H_2_O, purified by HPLC, lyophilized and characterized by HPLC and MALDI-TOF MS. For the synthesis of **M8**, to a solution of d-glutamic acid (0.30 g, 2.04 mmol) in 6.25 mL dry methanol, 0.89 mL distilled thionyl chloride (1.46 g, 12.23 mmol) was added at 0 °C over 30 min. Then, the reaction mixture was stirred for 12 h at room temperature. The solvent was evaporated under reduced pressure, diluted with saturated NaHCO_3_, and extracted with dicloromethane (3 × 200 mL). The organic layer was washed with H_2_O, followed by brine, dried over Na_2_SO_4_ and filtered. After evaporation of the solvent, the desired d-glutamic acid dimethyl ester was obtained as a yellowish oil (0.315 g, 1.79 mmol, 87% yield) [47]. NMR spectrum was measured on a 400 MHz Avance 4 Neo (Bruker) spectrometer. As solvents for NMR deuterated chloroform (CDCl_3_) was used (Euriso-top^®^). The chemical shifts (δ) were referenced to tetramethylsilane or the solvent peak and were given in parts per million (ppm). Coupling constants (J) were reported in Hertz (Hz). The following descriptors for signals were used: s = singlet, t = triplet, q = quartet, m = multiplet. ^1^H-NMR (400 MHz, CDCL_3_ δ ppm): 3.66 (s, 3H, CH_3_), 3.61 (s, 3H, CH_3_), 3.43–3.39 (q, 1H, CH), 2.43–2.39 (t, 2H, CH_2_), 2.04– 1.75 (m, 2H, CH_2_) (Figure S3). To d-glutamic dimethylester (0.030 mg, 0.174 mmol) in a round-bottomed flask a mixture of DOTA-tris(tert-butylester) (0.05 g, 0.087 mmol), HATU (0.066 g, 0.174 mmol), HOAt (0.024 g, 0.174 mmol) in 3 mL DCM, adjusted to pH 8 with 35 µL DIPEA, was added and the solution was stirred overnight at room temperature. The reaction solution was evaporated and the protecting groups were removed by adding 3 mL 50% TFA in DCM at 60 °C for 24 h. After evaporation, the crude product was dissolved in 2 mL 50% ACN. After HPLC purification and lyophilization, the final product was characterized by HPLC and MALDI-TOF MS.

### 4.3. Radiolabeling and Characterization In Vitro

For labeling with lutetium-177, the DOTA-peptides (8–12 µg in 10 µL) were incubated with 10–30 µL [^177^Lu]LuCl_3_ solution (60–300 MBq in 0.05 M HCl) and a >1.2-fold volume of 0.4 M sodium acetate/0.24 M gentisic acid solution pH adjusted to 5 at 90 °C for 20 min. Radiochemical purity of the radiopeptides was analyzed using the analytical HPLC system described above using a flow rate of 1 mL/min together with the following water/ACN/0.1% TFA gradient: 0–3 min 10% ACN, 3–18 min 10–55% ACN, 18–20 min 55–80% ACN, 20–21 min 80–10% ACN, 21–25 min 10% ACN. [^177^Lu]Lu-**1** used in animal studies was purified by solid phase extraction (SPE). For this purpose, a C18 SepPak tLight cartridge (Waters, Milford, MA) was pretreated with 5 mL 99% ethanol followed by 5 mL 0.9% saline. The radiolabeling solution was passed through the cartridge and washed with 5 mL 0.9% saline to elute hydrophilic impurities. The radiolabeled peptide was eluted with 50% ethanol from the cartridge and diluted with 0.9% saline.

For the determination of the distribution coefficient (LogD) in octanol/PBS, the radiolabeled DOTA-peptides (100 pmol) in 500 µL PBS (pH 7.4) were added to 500 µL octanol in an Eppendorf microcentrifuge tube (*n* = 8). The mixture was vigorously vortexed at room temperature over a period of 15 min using a small shaker (MS3 Basic, IKA, Staufen, Germany) with speed of 1500 rpm. After a waiting time of 10 min sufficient for the separation of the two phases, 100 µL aliquots of both layers were measured in a gamma counter (2480 Wizard2 3”, PerkinElmer Life Sciences and Analytical Sciences, formerly Wallac Oy, Turku, Finland) and the logD value was calculated.

For protein binding assessment, the radiolabeled DOTA-peptides were incubated in fresh human serum at 37 °C (500 pmol/mL, *n* = 2). After 1, 4 and 24 h of incubation, two samples were taken for each time point and analyzed by Sephadex G-50 size-exclusion chromatography (GE Healthcare Illustra, Little Chalfont, UK). The percentage of protein binding was determined by measuring the column and the eluate with the gamma-counter.

In vitro stability studies for the characterization of the metabolic stability of the radiolabeled peptide analogs in human serum were carried out with [^177^Lu]Lu-**1**, [^177^Lu]Lu-**M1**, [^177^Lu]Lu-**M2**, [^177^Lu]Lu-**M3** and [^177^Lu]Lu-**M4** (*n* = 2). Additional stability studies in rat liver and kidney homogenates were performed for [^177^Lu]Lu-**1** (*n* = 2). Tissue homogenates were prepared from dissected organs by homogenization for 1 min at RT (IKA-Werke, Staufen, Germany) in 20 mM HEPES buffer pH 7.3 (30% *v/v*). The radioligands were incubated in the different media at a concentration of 500 pmol/mL (corresponding to an activity of 6–11 MBq). After incubation at 37 °C at different time points for up to 24 h, a 100 µl sample was taken in duplicates and analyzed by HPLC. Samples obtained from human serum and rat homogenates were treated with ACN at a ratio of 1:1.5 (*v/v*) to precipitate proteins, centrifuged (14,000 rpm, 2 min, centrifuge 5424, Eppendorf AG, Germany) and diluted with water at a ratio of 1:1 (*v/v*). A 100 µl aliquot of this solution was injected into the radio HPLC system. The degradation of the radioligand was evaluated based on the radiochemical purity after radiolabeling and the percentage of intact radiopeptide observed during incubation in the different media.

### 4.4. Receptor Binding and Cell Uptake Studies

The binding affinity of **1,** pentagastrin and of the different metabolites for the CCK2R was tested in a competition assay against [^125^I][3-iodo-Tyr^12^,Leu^15^]gastrin-I. Radioiodination of gastrin-I was carried out using the chloramine-T method. [^125^I][3-iodo-Tyr^12^,Leu^15^]gastrin-I at high molar activity was obtained by HPLC purification and stored in aliquots at −25 °C. Binding assays were carried out using 96-well filter plates (MultiScreen_HTS_-FB, Merck Group, Darmstadt, Germany) pre-treated with 10 mM TRIS/139 mM NaCl pH 7.4 (2 × 250 µL). For the assay, 200,000–400,000 A431-CCK2R cells per well were prepared in 20 mM HEPES buffer pH 7.4 containing 10 mM MgCl_2_, 14 µM bacitracin and 0.5% BSA. The cells (*n* = 3) were incubated with increasing concentrations of the peptide conjugates (0.0003–30,000 nM) and [^125^I][3-iodo-Tyr^12^,Leu^15^]gastrin-I (50,000 cpm) for 1 h at RT. Incubation was interrupted by filtration of the medium and rapid rinsing with ice-cold 10 mM TRIS/139 mM NaCl pH 7.4 (2 × 200 µL) and the filters were measured in the gamma-counter. Half maximal inhibitory concentration (IC_50_) values were calculated following nonlinear regression with Origin software (Microcal Origin 6.1, Northampton, MA, USA).

For internalization experiments with [^177^Lu]Lu-**1** and its metabolites, A431-CCK2R and A431-mock cells were seeded at a density of 1.0 × 10^6^ per well in 6-well plates (Greiner Labortechnik, Kremsmuenster, Austria)and grown for 48 h until reaching almost confluence. On the day of the experiment, the medium was replaced by 1.2 mL of fresh DMEM medium supplied with 1% (*v/v*) fetal bovine serum. The cells (*n* = 3) were incubated with ~25,000 cpm of the radioligand in 300 µL PBS/0.5% BSA in a total volume of 1.5 mL, corresponding to a final concentration of 0.4 nM of total peptide. After 1 h and 2 h incubation, the cell uptake was interrupted by removal of the medium and rapid rinsing with 2 × 1 mL PBS/0.5% BSA. Thereafter, the cells were incubated twice at ambient temperature with acid wash buffer (50 mM glycine buffer pH 2.8, 0.1 M NaCl) for 5 min, to remove the membrane-bound radioligand. Finally, the cells were lysed by treatment in 1 N NaOH and collected (internalized radioligand fraction). All fractions were counted in the gamma-counter and mean values were calculated. The internalized fraction was expressed in relation to the total radioactivity added to the cells. In an additional experiment, the cell uptake of [^177^Lu]Lu-**M1**, [^177^Lu]Lu-**M2**, [^177^Lu]Lu-**M3** and [^177^Lu]Lu-**M4** was investigated in A431-CCK2R cells for the time point of 1 and 2 h incubation.

### 4.5. Metabolic Biodistribution Studies and Characterization of the Radiometabolites In Vivo

Metabolic biodistribution studies were performed in accordance with the ethical standards of the institution and approved by the Austrian Ministry of Science (BMWFW-66.011/0072-V/3b/2019). These studies were carried out in 5- to 7-week-old female BALB/c mice (*n* = 3) injected with [^177^Lu]Lu-**1** via a lateral tail vein. To allow monitoring of the metabolites by radio-HPLC, mice were injected with 30 MBq corresponding to 0.8 nmol total peptide. The mice were euthanized by cervical dislocation after different time points of 10 min, 30 min and 1 h p.i. and the urine and a venous blood sample were collected at the time of sacrifice. Liver and kidneys were dissected and homogenized in 20 mM HEPES buffer pH 7.3 at a ratio of 1:1 (*v/v*) with an Ultra-Turrax T8 homogenizer (IKA-Werke, Staufen, Germany) for 1 min at RT. Before radio-HPLC analysis, the samples (except urine) were treated with ACN at a ratio of 1:1 (*v/v*) to precipitate proteins, centrifuged at 2000× *g* for 2 min and diluted with water at a ratio of 1:1 (*v/v*). For HPLC analysis of the samples, the analytical HPLC system described above with a flow rate of 1 mL/min was used together with the following optimized water/ACN/0.1% TFA gradient to allow a better separation of [^177^Lu]Lu-**1** and the different radiometabolites: 0–7 min 1–7% ACN, 7–8 min 7–10% ACN, 8–18 min 10–18% ACN, 18–20 min 18–32% ACN, 20–27 min 32% ACN, 27–37 min 32–0% ACN, 37–40 min 80% ACN, 40–40.1 min 80–1% ACN, 40.1–45 min 1% ACN. Urine was measured with the low-sensitivity loop of the radiodetector. The remaining samples were analyzed with the high-sensitivity loop due to the lower radioactivity present in blood, liver and kidney homogenate. To quantify the percentage of intact [^177^Lu]Lu-**1** and [^177^Lu]Lu-**M1** showing some overlap in the radiochromatogram when using the high-sensitivity loop of the radiodetector, the two radiopeptides were co-injected at different known ratios to enable a more accurate separation and integration of the two peaks (see supporting Figure S4). Mice from the metabolic biodistribution studies were subjected to a further dissection of all remaining tissues (blood, lung, heart, femur, spleen, muscle, intestine, pancreas and stomach). All organs, also including liver and kidneys, were weighed, and their radioactivity was measured in the gamma counter together with a standard and the residual body. To quantify the uptake of radioactivity in liver and kidneys, a part of liver and kidney homogenate was measured in the gamma counter to extrapolate the radioactivity for the whole organ.

## 5. Conclusions

Radiolabeled MG analogs with the modified receptor-specific C-terminal sequence Trp-(*N*-Me)Nle-Asp-1Nal-NH_2_ are promising new candidates for diagnostic and therapeutic use in patients with advanced MTC and other CCK2R-expressing malignancies. [^177^Lu]Lu-**1** with introduction of an additional tertiary peptide trough substitution with Pro in position 2, shows a highly improved stability against enzymatic degradation in vivo. From the radiometabolites identified in the blood of mice injected with [^177^Lu]Lu-**1** hydrolysis of the C-terminal amide and cleavage of the peptide bonds of Asp-1Nal, (*N*-Me)Nle-Asp and Gly-Trp were found to occur in vivo. The high receptor-mediated cell uptake and favorable biodistribution profile in normal BALB/c mice support further studies evaluating the tumor targeting potential of [^177^Lu]Lu-**1** and other alternative derivatives thereof.

## Figures and Tables

**Table 1:** Summary of the analytical data of **1** and the metabolites **M1**–**M8** (radio t_R_ obtained after labeling with lutetium-177).

Peptide	Peptide Sequence	Purity(%)	Radio t_R_ [min]	UV t_R_ [min]	MW calc *m/z* [M + H]^+^	MW found *m/z* [M + H]^+^
**1**	DOTA-DGlu-Pro-Tyr-Gly-Trp-(*N*-Me)Nle-Asp-1Nal-NH_2_	>95	32.6	32.4	1475.6	1475.8
**M1**	DOTA-DGlu-Pro-Tyr-Gly-Trp-(*N*-Me)Nle-Asp-1Nal-COOH	94	33.1	32.8	1476.6	1475.9
**M2**	DOTA-DGlu-Pro-Tyr-Gly-Trp-(*N*-Me)Nle-Asp	99	24.9	24.9	1278.4	1279.2
**M3**	DOTA-DGlu-Pro-Tyr-Gly-Trp-(*N*-Me)Nle	98	25.8	26.0	1163.3	1164.2
**M4**	DOTA-DGlu-Pro-Tyr-Gly-Trp	93	23.5	23.4	1036.1	1037.4
**M5**	DOTA-DGlu-Pro-Tyr-Gly	99	13.7	15.2	849.9	851.4
**M6**	DOTA-DGlu-Pro-Tyr	96	14.4	16.7	792.8	794.5
**M7**	DOTA-DGlu-Pro	99	9.8	12.6	630.6	631.5
**M8**	DOTA-DGlu	99	6.3	6.4	533.5	534.5

**Table 2:** Biodistribution profile of [^177^Lu]Lu-**1** in BALB/c mice after 10 min, 30 min and 1 h p.i. (30–40 MBq, 0.8 nmol). Values are expressed as % IA/g.

Organ	% IA/g		
	10 min p.i.	30 min p.i.	1 h p.i.
blood	17.79	5.08	2.05
lung	17.45	5.03	1.95
heart	7.13	2.72	0.99
femur	5.97	1.28	0.52
spleen	3.70	2.00	0.79
muscle	2.56	1.03	0.36
intestine	2.63	1.61	1.02
liver	5.37	2.95	1.26
kidneys	14.49	10.76	7.01
pancreas	3.39	1.76	0.84
stomach	3.36	2.29	1.39

**Table 3:** Quantification of the percentage of intact radiopeptide and of the radiometabolites as analyzed by radio-HPC of blood and urine, as well as liver and kidneys of BALB/c mice injected with [^177^Lu]Lu-**1** for different time points p.i.

Peptide	Blood		Liver		Kidney		Urine	
	30 min	60 min	30 min	60 min	30 min	60 min	30 min	60 min
[^177^Lu]Lu-**1**	64.1%	56.9%	53.9%	47.7%	18.0%	7.0%	19.4%	18.0%
[^177^Lu]Lu-**M1**	9.9%	5.8%	4.8%	4.6%	2.5%	-	1.4%	-
[^177^Lu]Lu-**M2**	3.8%	7.3%	7.2%	6.9%	20.7%	12.3%	40.5%	40.3%
[^177^Lu]Lu-**M3**	17.0%	20.4%	8.5%	5.6%	7.9%	-	9.5%	9.8%
[^177^Lu]Lu-**M4**	-	-	2.9%	2.6%	-	-	-	-
[^177^Lu]Lu-**M5**	5.3%	9.6%	14.4%	12.9%	27.3%	23.7%	28.7%	31.3%
[^177^Lu]Lu-**M6**	-	-	8.3%	19.7%	23.6%	53.2%	-	-
[^177^Lu]Lu-**M7**	-	-	-	-	-	3.8%	-	-
[^177^Lu]Lu-**M8**	-	-	-	-	-	-	-	-

## Supplementary Materials

The following are available online, Figure S1: Representative UV-chromatogram of the metabolites **M1**–**M8**; Figure S2: MALDI-TOF-MS of the different synthesized metabolites **M1**–**M8**; Figure S3: 400 MHz 1H NMR of D-glutamic acid dimethyl ester; Figure S4. Radiochromatograms of [^177^Lu]Lu-**1** and [^177^Lu]Lu-**M1** co-analyzed in different ratios of approximately (a) 1:1, (b) 2:1 and (c) 10:1 (*v/v*) using the radiodetector equipped with the high sensitivity loop (250 µL)
