# Supplementary material for: Initial In Vitro and In Vivo Evaluation of a Novel CCK2R Targeting Peptide Analog Labeled with Lutetium-177
Source: Molecules. 2020 Oct 8;25(19):4585. doi: 10.3390/molecules25194585 (PMC7583830; doi:10.3390/molecules25194585)
Supplement: Supplementary file 1 [file molecules-25-04585-s001.pdf]

Initial In Vitro and In Vivo Evaluation of a Novel CCK2R Targeting Peptide Analog Labeled with Lutetium-177

Anton Amadeus Hörmann [1], Maximilian Klingler [1], Maliheh Rezaeianpour [1,3], Nikolas Hörmann [2], Ronald Gust [2], Soraya Shahhosseini [3] and Elisabeth von Guggenberg [1,*]

[1] Department of Nuclear Medicine, Medical University of Innsbruck, 6020 Innsbruck, Austria; anton.hoermann@i-med.ac.at (A.A.H.); Maximilian.Klingler@i-med.ac.at (M.K.); m.rezaeianpour@yahoo.com (M.R.)

[2] Department of Pharmaceutical Chemistry, University of Innsbruck, 6020 Innsbruck, Austria; nikolas.hoermann@uibk.ac.at (N.H.); ronald.gust@uibk.ac.at (R.G.)

[3] Pharmaceutical Chemistry and Radiopharmacy Department, School of Pharmacy, Shahid Beheshti University of Medical Sciences, 1991953381 Tehran, Iran; soraya.shahhosseini@gmail.com

* Correspondence: elisabeth.von-guggenberg@i-med.ac.at; Tel.: +43-512-504-80960

Table of Contents

Figures S. 2

Figure S1: Representative UV-chromatogram of the metabolites **M1**-**M8**
Figure S2: MALDI-TOF-MS of the different synthesized metabolites **M1**-**M8**
Figure S3: 400 MHz ^{1}H NMR of D-glutamic acid dimethyl ester
Figure S4: Representative radiochromatogram of [^{177}Lu]Lu-**1** and [^{177}Lu]Lu-**M1** co-analyzed in different ratios of approx. 1:1 (a) 2:1 (b) and 10:1 (c) using the radiodetector equipped with the high sensitivity loop (250 µL)

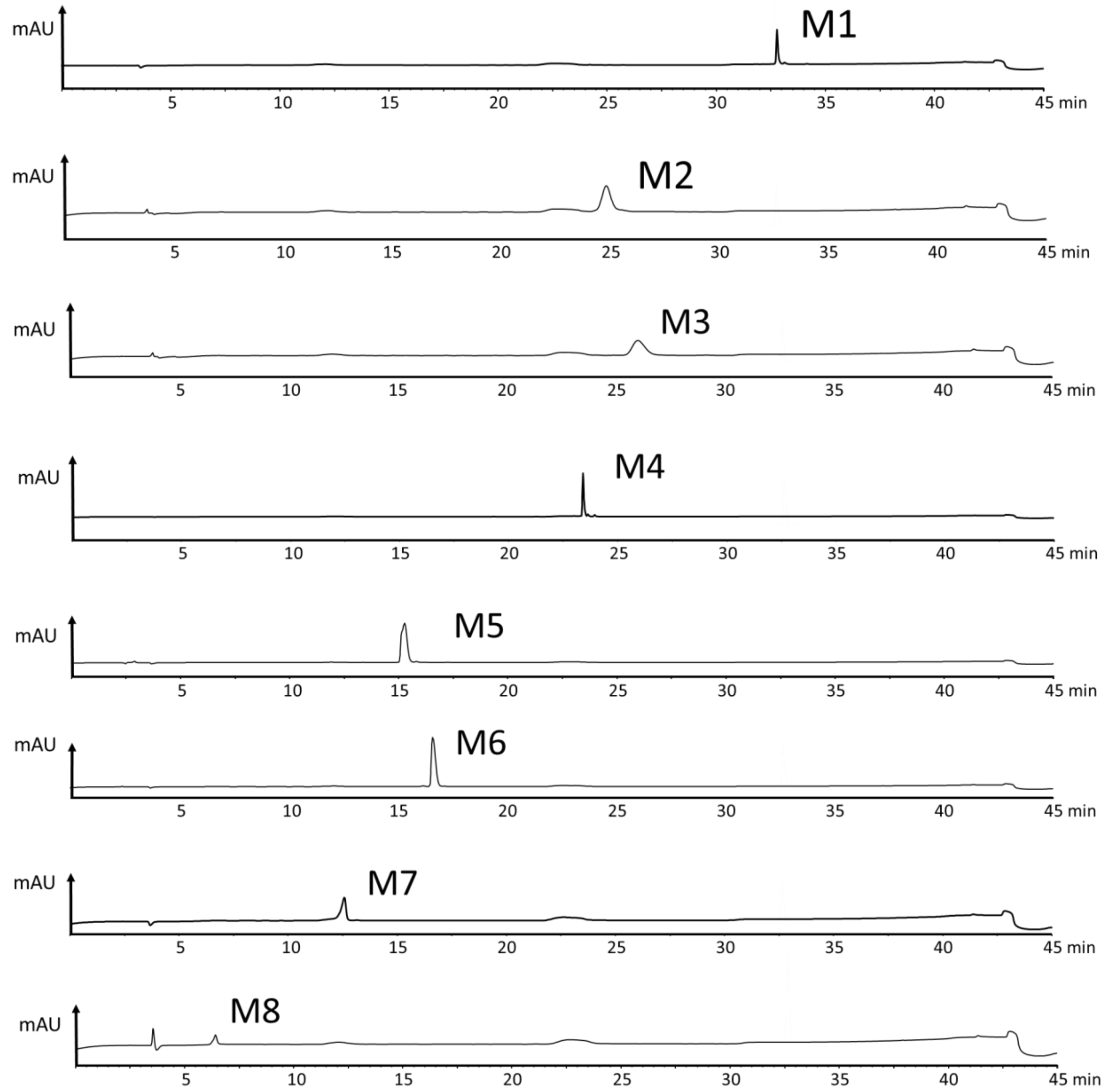

Figure S1: Representative UV-chromatogram of the metabolites **M1**-**M8**

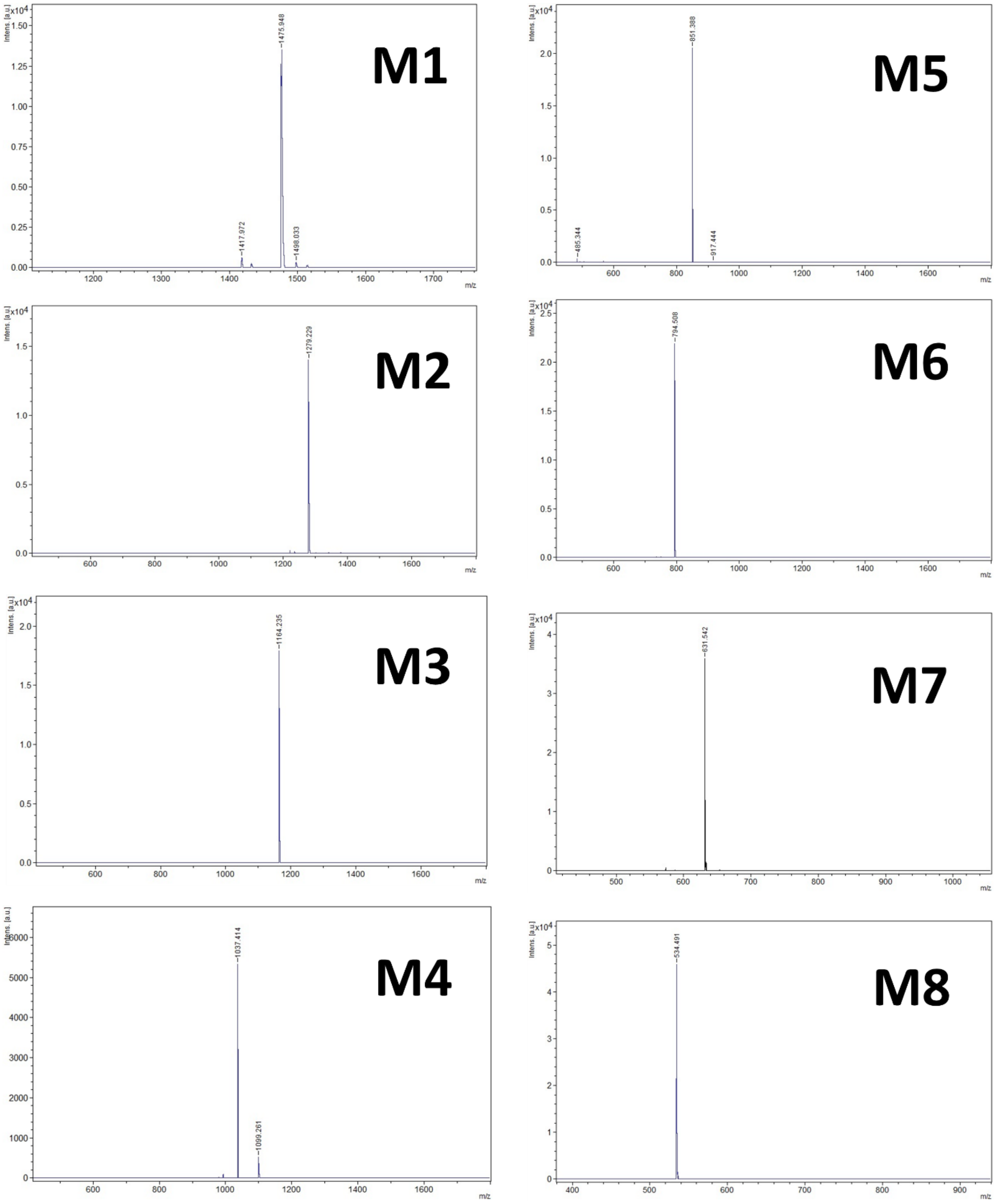

Figure S2: MALDI-TOF MS spectra of the synthesized metabolites **M1-M8**

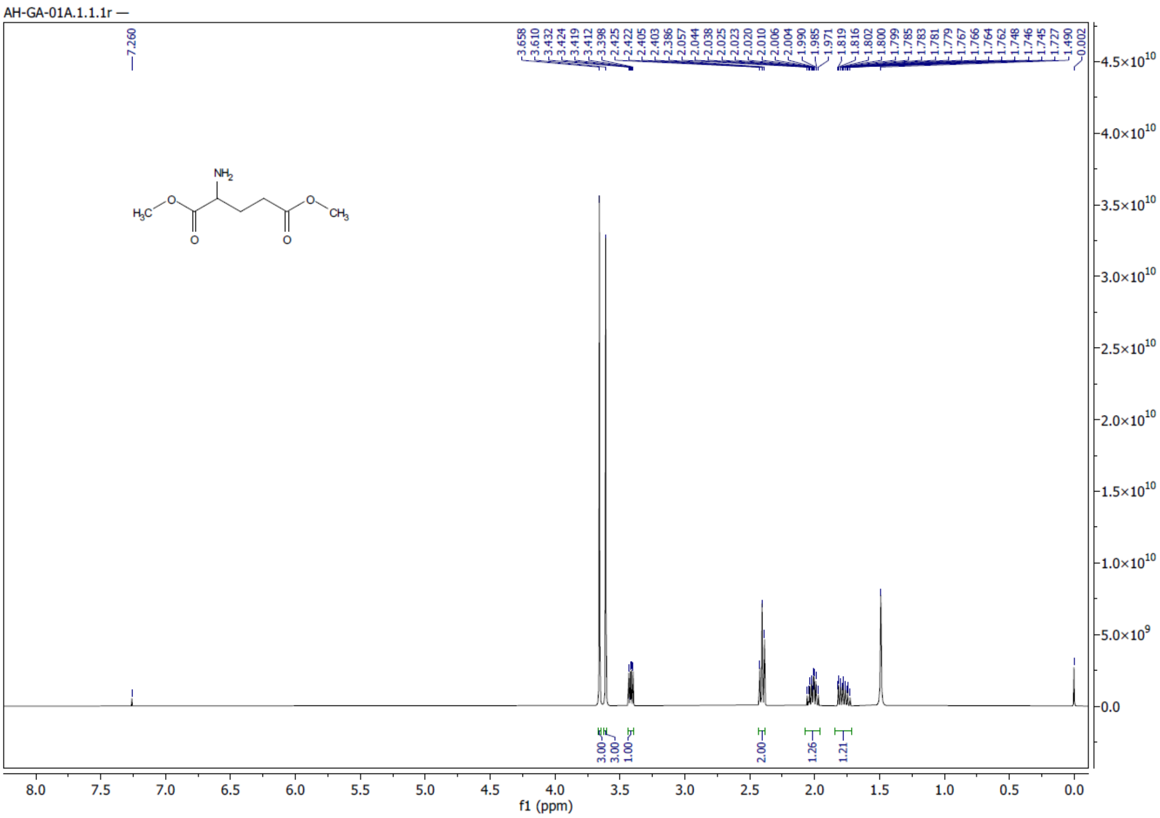

Figure S3: 400 MHz ^{1}H NMR of **D-glutamic acid dimethyl ester**

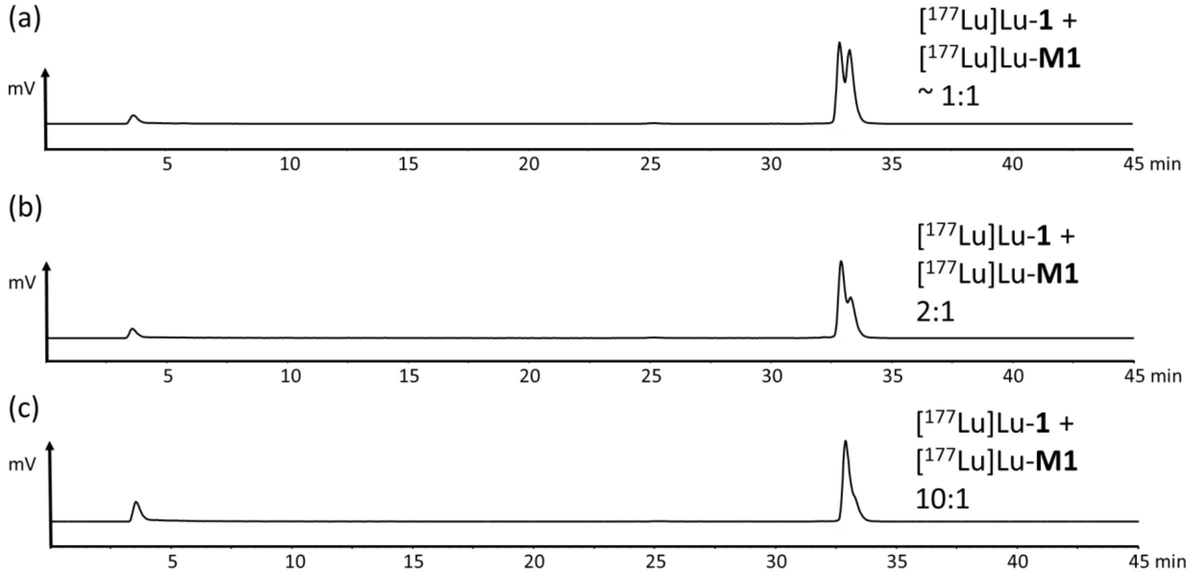

Figure S4: Representative radiochromatogram of [^{177}Lu]Lu-**1** and [^{177}Lu]Lu-**M1** co-analyzed in different ratios of approx. 1:1 (a) 2:1 (b) and 10:1 (c) using the radiodetector equipped with the high sensitivity loop (250 µL)
